# Intervention and Public Policy Pathways to Achieve Health Care Equity

**DOI:** 10.3390/ijerph16142465

**Published:** 2019-07-11

**Authors:** Shelley White-Means, Darrell J. Gaskin, Ahmad Reshad Osmani

**Affiliations:** 1 Department of Interprofessional Education, College of Graduate Health Sciences, University of Tennessee Health Science Center, Memphis, TN 38163, USA; 2 CHEER Health Disparities Center, University of Tennessee Health Science Center, Memphis, TN 38163, USA; 3 Department of Health Policy and Management, Johns Hopkins Bloomberg School of Public Health, Baltimore, MD 21205, USA; 4 Hopkins Center for Health Disparities Solutions, Johns Hopkins Bloomberg School of Public Health, Baltimore, MD 21205, USA; 5 Department of Economics, University of Memphis, Memphis, TN 38152, USA

**Keywords:** health care equity, NIMHD framework, interventions, public policy

## Abstract

Health care equity reflects an equal opportunity to utilize public health and health care resources in order to maximize one’s health potential. Achieving health care equity necessitates the consideration of both quantity and quality of care, as well as vertical (greater health care use by those with greater needs) and horizontal (equal health care use by those with equal needs) equity. In this paper, we summarize the approaches introduced by authors contributing to this Special Issue and how their work is captured by the National Institute of Minority Health and Health Disparities (NIMHD) framework. The paper concludes by pointing out intervention and public policy opportunities for future investigation in order to achieve health care equity.

## 1. Introduction

Health care equity reflects an equal opportunity to utilize public health and health care resources in order to maximize one’s health potential [1]. This potential requires equitable access to and use of preventive, diagnostic, and therapeutic services. Achieving health care equity necessitates the consideration of both quantity and quality of care, as well as vertical (greater health care use by those with greater needs) and horizontal (equal health care use by those with equal needs) equity [2]. Health care inequities may occur due to disparities in distal, transitional and proximal causes that influence health care outcomes [3]. Distal causes refer to socio-environmental factors, e.g., transportation systems, exposure to pollutions, and availability of health care providers. Transitional causes are factors that are closer to health care outcomes such as individual health behaviors. Proximal causes are the biological factors that cause disease and hinder or facilitate recovery [4,5,6]. Government officials, health care providers, community leaders, academic researchers and patients have been working on policies, interventions, and strategies to address health care inequity.

This special issue of the International Journal of Environmental Research and Public Health documents how recent or proposed policy changes and interventions affect health care equity, with emphasis on the roles of how biology, behavior, the physical/built environment, the sociocultural environment, and the health care system influence at the individual, interpersonal, community, and societal levels. Lessons learned across health care settings, geographic regions, and underserved populations are provided. The National Institute of Minority Health and Health Disparities (NIMHD) provides a research framework for understanding the multifaceted domains of influence and levels of influence within domains that facilitate changes in health care equity [7]. In this paper, we summarize the approaches introduced by authors contributing to this Special Issue (Appendix A) and how their work is captured by the NIMHD framework.

## 2. NIMHD Framework

The NIMHD health disparities framework (Table 1) provides a way to conceptualize the multiple ways that various categories of factors may influence health outcomes, leading to reductions or improvements in health equity [7]. It builds on the National Institute on Aging (NIA) health disparities research framework [8] by combining it with the socio-ecological model developed by Urie Brofenbrenner [9]. The NIA framework focused on the four domains that influence health disparities (biological, behavioral, environmental, and sociocultural) and NIMHD added an additional domain of health systems. Bronfenbrenner’s socioecological model conceptualized that health and human development are affected at multiple levels. These levels are individual, interpersonal (including family, school, work, social networks, churches, and health services providers), community (e.g., neighbors, social services, industry, mass media, local politics), and societal (attitudes and ideologies of the culture, policies and laws of states, regions of the country or the nation). NIMHD combined these two frameworks to form a 20-cell matrix (Table 1) where each cell describes a determinant of health equity.

## 3. Special Issue Emphasis

Articles in this Special Issue primarily focus on two domains of influence in the NIMHD framework, i.e., sociocultural environment and the health care system. All levels of influence are considered, especially community and societal influences.

### 3.1. Health Equity Solutions from a Health Care Systems Approach

Considering influences at the individual level, White-Means and Osmani [10] report racial inequities in breast cancer mortality can be reduced by comprehensive health insurance coverage providing preventive care access and lower out-of-pocket costs. Wang et al. [11] also report that emphasis on increasing health insurance and patients’ knowledge about self-health would reduce inequities in patient experiences with their hospital providers.

Considering influences at the community level, Mon Kyaw Soe et al. [12] note that preventive interventions that focused on sexually transmitted infection and were administered in educational settings had a significantly positive impact on both behavioral and psychosocial outcomes; they were most effective at promoting knowledge, enhancing motivational factors, and improving behavioral skills. Yu et al. [13] report that implementing a report card program for patients undergoing total knee replacement surgery increased inequities between urban higher income and rural lower income patients because hospital selection varied based on socioeconomic status.

Considering influences at the societal level, Zhu et al. [14] use spatial analysis to posit that methods are needed to redefine equity in provider distribution, which is currently measured by equal access to providers, although some communities need greater access than others. In essence, current strategies should focus on vertical equity. In public policy planning aimed at health care equity, Wu and Tseng [15] note the importance of using geographic information system (GIS) to jointly assess geographic accessibility and equality of resource allocation, e.g., travel distance and whether travel is by public transportation or car. Population demand and supplier capacity should also be incorporated in planning. Sándor et al. [16] proposed revising human resource policy in health care systems, i.e., retirement policy, so that sufficient providers are available to forestall increases in premature mortality when provider shortages exist. Nanney et al. [17] posit that creating a climate for health system change can be based on assessing the cost of not making a change. They report that in Minnesota, if racial disparities in preventable deaths were eliminated, 475 to 812 lives would be saved each year, generating a financial savings of $1.2 billion to $2.9 billion. 

### 3.2. Health Equity Solutions from a Sociocultural Environment Approach

Demeke et al. [18] note that sociocultural environments may influence equity in the time to and stage of treatment, as well as outcomes. They note that care must be taken in understanding differences by race and country of origin. Thus, to understand and design needed human immunodeficiency virus (HIV) prevention and control programs, one must be careful not to merge strategies developed for US-born and non-US-born blacks as though individuals in these demographic categories belong to the same group; they are not in the same group and differ in their protective characteristics. While patient–provider concordance has been a strategy for improving health outcomes, Oguz [19] found that Hispanic men have greater satisfaction with their health care when the provider is non-Hispanic. Wang et al. [20] emphasize that along with valuing clinically competent primary care health professionals, patients also value being treated respectfully and receiving clear communications from their physicians.

Considering influences at the community and societal level, Smith et al. [21] note that implementing community-based participatory research strategies, where community trust agents are engaged and community education on disease states is utilized, provide an opportunity to reach underserved populations and enhance their health outcomes. Social context is important in understanding racial disparities in physical activity [22]. The authors report that individual poverty and neighborhood poverty are associated with decreased odds of being physically active among both whites and blacks. He et al. [23] note that reducing wealth inequality among women should be a focus of national public health programs aimed at improving women’s health and well-being. However, the strategies for implementing such a policy must take into consideration existing sociocultural conditions mediating the role of household wealth status on women’s lives, such as deep-rooted gender inequality in the social value system.

### 3.3. Health Equity Solutions from a Physical/Built Environment Approach

Considering influences at the community level, Gaskin et al. [24] quantify the role of neighborhood disadvantage and find that the hazard of dying increased by 9.8% as neighborhood disadvantage increased by one standard deviation. Area-level poverty and mortgage delinquency were important predictors of mortality, even after controlling for individual personal income and occupational status.

### 3.4. Health Equity Solutions from a Behavioral Approach

Considering societal influences, Nolasco et al. [25] report geographical inequalities between provinces, both in mortality rates and avoidable mortality rates, that may be explained by inequalities in the political management of the crisis that occurred in each of the provinces. They also note that researchers must take caution in reporting possible associations between mortalities and economic crises because these factors are pro-cyclical, i.e., mortality rates are lower during economic crises and higher during economic recovery. Short-term data may not capture long-term associations.

### 3.5. Health Equity Solutions from a Three Component Approach that Combines the Physical/Built Environment, Sociocultural Environment, and Health Care System Approaches

Williams and Cooper [26] are unique in their approach to resolving health care inequities. In contrast to other authors, they maintain that eliminating racial inequities in health care requires a three-fold coordinated strategy. The three parts are: (1) addressing racism that incorporates place-based barriers to resources needed to enhance access, (2) health care systems that are culturally competent and emphasize prevention, and (3) knowledge, empathy and political will to eliminate racial inequities in care.

The articles in this Special Issue suggest a number of avenues to achieve health care equity. All of these avenues are very much in keeping with the NIMHD framework. Some require us to address needed change from the perspective of individual patients, while others require changes in interpersonal relations, and community and societal perspectives. Health equity is complicated. There is no single policy, program or intervention that will remedy inequities in health care. Health equity is a value that must permeate all that we do in health care and similar to access, quality and efficiency, equity must be continuously pursued. These articles suggest that the pursuit of health equity must be dynamic, multifaceted and multilayered.

## 4. Conclusions

Overall, this Special Issue of IJERPH identifies several strategies combining domains and levels of influence that can potentially enhance health equity. It also points out opportunities for future investigation. When NIMHD matched its 2015 awards with the NIMHD health disparities framework, it noted that several cells of the matrix were unfunded [7]. These included the biological domain as influenced by community level and societal factors. Similarly, very limited funding has been provided for research that focuses on community and societal level influences across all domains. Across domains, the least likely to receive funding by NIMHD is research that focuses on the physical/built environment. These are all areas of opportunity for providing new insights on strategies to enhance health equity.

## Figures and Tables

**Table 1:** National Institute on Minority Health and Health Disparities’ Health and Health Disparities Research Framework.

		Levels of Influence *			
		Individual	Interpersonal	Community	Societal
**Domains of Influence** *(Over the Life Course)*	**Biological**	Biological Vulnerability and Mechanisms	Caregiver–Child Interaction Family Microbiome	Community Illness Exposure Herd Immunity	Sanitation Immunization Pathogen Exposure
	**Behavioral**	Health Behavior Coping Strategies	Family Functioning School/Work Functioning	Community Functioning	Policies and Laws
	**Physical/Built Environment**	Personal Environment	Household Environment School/Work Environment	Community Environment Community Resources	Societal Structure
	**Sociocultural Environment**	Sociodemographics Limited English Cultural Identity Response to Discrimination	Social Networks Family/Peer Norms Interpersonal Discrimination	Community Norms Local Structural Discrimination	Social Norms Societal Structural Discrimination
	**Health Care System**	Insurance Coverage Health Literacy Treatment Preferences	Patient–Clinician Relationship Medical Decision-Making	Availability of Services Safety Net Services	Quality of Care Health Care Policies
**Health Outcomes**		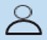 **Individual Health**	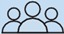 **Family/Organizational** **Health**	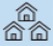 **Community Health**	**Population Health**

National Institute on Minority Health and Health Disparities, 2018. * Health Disparity Populations: Race/Ethnicity, Low SES, Rural, Sexual/Gender Minority Other Fundamental Characteristics: Sex/Gender, Disability, and Geographic Region.

## Appendix A. Article Summaries and Categorization

### Reducing Racial Inequities in Health: Using What We Already Know to Take Action

Domain of Influence: Health Care System; Built Environment; Sociocultural Environment

Level of Influence: Societal; Community; Community Quality of Care and Health Care Policy; Community Resources; Community Norms and Local Structural Discrimination

The paper by Williams and Cooper [26] emphasizes the role of available scientific evidence in reducing racial and ethnic disparities in health care systems. Specifically, needed are insights on resources available for communities and responsive health care systems that could address the health care needs of society at broader levels and strengthen targeted research that provides policy and strategic directions to reduce system-level health care disparities. These steps could ultimately improve the quality and quantity of health care delivery at the societal level. Political willingness is the key and the precursor for actionable implementation of any of this known evidence.

### STI [Sexually Transmitted Infection] Health Disparities: A Systematic Review and Meta-Analysis of the Effectiveness of Preventive Interventions in Educational Settings

Domain of Influence: Health Care System

Level of Influence: Community Availability of Services and Safety Net Services

Availability of quality and equitable health care services for sufferers of sexually transmitted diseases (STDs) is the key public health policy agenda in both developed and developing countries. The systemic review and meta-analysis by Mon Kyaw Soe, Bird, Schwandt and Moraros [12] indicate the role of preventive strategies in changing the behavioral and psychological factors that determine the effectiveness of policy planning, implementation and evaluation for STD patients at educational institutions.

### Incorporating Spatial Statistics into Examining Equity in Health Workforce Distribution: An Empirical Analysis in the Chinese Context

Domain of Influence: Health Care System

Level of Influence: Societal Quality of Care

Both horizontal and vertical health equity are the key public health issues in developed and developing countries. However, methodological shortcomings in measuring health equity have limited the broader understanding and interpretation of traditional economic methods. For example, it is hard to prioritize the equitable distribution of health labor forces based upon the recommendations of economic methods that solely provide directions on equal distribution of health labor forces. The paper by Zhu, Hsieh and Zhang [14] introduces the execution of spatial analysis in analyzing equity in the health labor force. The later mentioned method is a new paradigm in understanding the interpretation of traditional tools of health equity analysis.

### Evaluating Disparities in Elderly Community Care Resources: Using a Geographic Accessibility and Inequality Index

Domain of Influence: Health Care System

Level of Influence: Societal Health Care Policies

Allocation of available resources to improve both the quality and quantity of health care services for the elderly population has been a top health policy agenda in most countries. Identifying the factors that determine the rational distribution of equitable health care resources (e.g., supply and demand-sides) requires application of broadly defined and inclusive methods. By applying ESRI’s ArcGIS “Model Builder” programming method to develop a domain partition origin-destination cost matrix calculation approach, Wu and Tseng [15] examined inequality in accessibility to community-based health care resources among elder population of Taiwan. Their study could inform policy in defining geographic accessibility while taking into consideration both demand and supply side factors.

### Association Between the General Practitioner Workforce Crisis and Premature Mortality in Hungary: Cross-Sectional Evaluation of Health Insurance Data from 2006 to 2014

Domain of Influence: Health Care System

Level of Influence: Societal Quality of Care/Health Care Policy

Lack of sufficient human resources in the health care sector has reached crisis in most parts of the world. Although the intensity of the crisis varies across regions, the problem in some countries is so grave that it requires urgent action. Studies have recognized a complex set of factors that has contributed to this problem. However, there is less understanding about the age distribution of the health labor force and its possible affects on overall health outcomes of a population. Using the Hungarian cross-sectional data for 2006–2014, Sándor, Pálinkás, Vincze, Sipos, Kovács, Jenei, Falusi, Pál, Kőrösi and Papp [16] evaluated the facilitation of labor market opportunities for older general practitioners and its positive affects on lowering premature mortality at primary health care facilities. Their study could motivate policy regarding the retirement age of medical doctors who are primarily responsible for the provision of health care services at primary health care facilities.

### Can Urban–Rural Patterns of Hospital Selection Be Changed Using a Report Card Program? A Nationwide Observational Study

Domain of Influence: Health Care System

Level of Influence: Community Level Availability of Services

Physician-related factors play a significant role in lowering health care disparities. Supporting patients to choose their preferred physician regardless of their socioeconomic and geographic locations could improve patient–physician communication and ultimately improve treatment outcomes. Using multivariate and logistic regression methods, Yu, Matthes and Wei [13] analyzed the impact of patient-level factors on hospital report cards. These cards provide a quality-focused scorecard for individual hospitals. Their study could determine how patient characteristics could impact the quality of services by health care institutions.

### Affordable Care Act and Disparities in Health Services Utilization Among Ethnic Minority Breast Cancer Survivors: Evidence from Longitudinal Medical Expenditure Panel Surveys 2008–2015

Domain of Influence: Health Care System

Level of Influence: Individual Insurance Coverage

Disparities in the use of health care services among cancer survivors of certain race and ethnicities in the US have been a highly debated public policy issue. Although medical technologies have changed the course and prognosis of cancers, there is still concern regarding the number of mortalities associated with cancer among non-Hispanic black and Hispanic Americans. Among others, a lack of access to needed health care services contributes to higher cancer-related mortalities among non-Hispanic and Hispanic Americans. To evaluate this issue, White-Means and Osmani [10] have hypothesized that inclusive and wide health care coverage could increase the utilization of health care services and lower the burden of out-of-pocket health expenditure for non-Hispanic blacks and Hispanics. Their study could help inform policy directions for the minority survivors of breast cancer who suffer the most from a lack of sufficient health care services.

### Economic Crisis and Amenable Mortality in Spain

Domain of Influence: Behavioral

Level of Influence: Societal Policies and Law

Socioeconomic-related inequality in the utilization of health care services could contribute to poor health outcomes. The problem could be triggered by an exogenous economic crisis. To evaluate the extent of inequality among sufferers of different health conditions and their associated mortalities during the economic recession, Nolasco, Pereyra-Zamora, Sanchis-Matea, Tamayo-Fonseca, Caballero, Melchor and Moncho [25] exploited Spanish datasets at a provincial level to investigate the effects of Spain’s economic downturn on avoidable mortalities. They cautiously concluded that there was a possible association between mortalities and economics crises as a result of pro-cyclical effects.

### HIV Infection-Related Care Outcomes Among US-Born and Non-US-Born Blacks with Diagnosed HIV in 40 US Areas: The National HIV Surveillance System, 2016

Domain of Influence: Sociocultural Environment

Level of Influence: Individual, Sociodemographic

Equitable access to health care services could be negatively affected by language barriers, lack of health insurance, pre-diagnosed chronic health conditions (e.g., HIV, cancer etc.) and immigration status for US residents born outside the US. Studies have indicated differences in health-related behaviors and physical and sociocultural environments between US born and non-US born individuals. Given these differences, it is important to separately evaluate the utilization related infections, where persons of different birth origins could disproportionately suffer from disparities in health outcomes. The analysis by Demeke, Johnson, Zhu, Gant, Duffus and Dean [18] evaluated these differences among black citizens who were not born in the US and compared their outcomes with US born black citizens. Their findings could inform policy in terms of heterogeneity in the health outcome of different socio-cultural and economic groups.

### Wealth Inequality as a Predictor of Subjective Health, Happiness and Life Satisfaction Among Nepalese Women

Domain of Influence: Sociocultural Environment

Level of Influence: Social Norms

Socioeconomic status could impact the health-related outcomes of communities. In most developing and developed countries, improvement in national health indicators is directly associated with the proper development and mobilization of monetary and non-monetary resources toward empowering social welfare and economic status. However, there is less understanding about the effects of social and economic norm changes and health outcomes in Nepal. Relying on a cross-sectional dataset of Nepalis women, He, Cheng, Bishwajit and Zou [23] studied the association between wealth inequality and subjective health outcomes. Their study indicates the strong predicting role of socioeconomic factors on health outcomes.

### Sistas Taking a Stand for Breast Cancer Research (STAR) Study: A Community-Based Participatory Genetic Research Study to Enhance Participation and Breast Cancer Equity Among African American Women in Memphis, Tennessee

Domain of Influence: Sociocultural

Level of Influence: Community Norms

The analysis by Smith, Vidal, Pritchard, Blue, Martin, Rice, Brown and Starlard-Davenport [21] described the importance of genetic research studies in evaluating cancer-related mortality among black breast cancer survivors in Memphis, Tennessee. Their study indicated that a community-based participatory approach could help recruit more breast cancer survivors in genetic research projects. Building trust and educating women through a participatory approach could contribute towards determining the differences in biological factors among black and white communities.

### Is Patient–Provider Racial Concordance Associated with Hispanics’ Satisfaction with Health Care?

Domain of Influence: Sociocultural Environment

Level of Influence: Interpersonal Discrimination

The study by Oguz [19] tested the hypothesis of patient–provider racial concordance for Hispanic Americans. Unlike the previous body of literature, this study concluded that Hispanic men achieve more satisfaction from their health care services when the provider is non-Hispanic. This study could improve policies relying on assumptions of concordance based on observable characteristics. It is recommended that further considerations should be given to minorities in terms of their provider selection.

### Public Hospitals in China: Is There a Variation in Patient Experience with In-Patient Care?

Domain of Influence: Health Care System

Level of Influence: Individual Insurance Coverage/Health Literacy

Patient experiences with health care services has been a key public policy agenda and significant determinant of health care quality. However, there is less understood about patient experiences at in-patient wards in a Chinese context. The study by Wang, Loban and Dionne [11] evaluated the variation in patient experience at in-patient wards between rural and urban China. Controlling for multiple confounding factors, they found that there is significant gap in patient experience in rural and urban China. Availability of health insurance and patient literacy on health-related outcomes could partly determine their experience with the providers at a hospital level.

### Perceived Impact of Taiwan’s National Health Insurance Allocation Strategy: Health Professionals’ Perspective

Domain of Influence: Health Care System

Level of Influence: Societal Quality of Care/Health Policy

Efficiency, sustainability and affordability of health insurance are the key components of health care systems in developed countries. These components have broader implications in lowering socioeconomic disparities in the utilization of health care systems. Perspectives of both demand and supply sides of the market could potentially improve the outcome of any health insurance and risk pooling mechanism. The current study by Owili et al. [27] evaluated the role of providers in designing the allocation criteria for the national health insurance fund.

### Determinants of Overall Satisfaction with Public Clinics in Rural China: Interpersonal Care Quality and Treatment Outcome

Domain of Influence: Sociocultural Environment

Level of Influence: Interpersonal Discrimination

Patient-centered health care service delivery is an integral part of quality health care provisions. Clinical improvement without taking interpersonal and patient–provider communication into consideration is hard to achieve. It is well-documented that lower interpersonal care qualities between patient and provider leads to misleading diagnoses and ultimately poor clinical outcomes. The study by Wang, Maitland, Nicholas and Haggerty [20] evaluated the effects of interpersonal communication on treatment outcomes in rural China.

### The Economic Benefits of Reducing Racial Disparities in Health: The Case of Minnesota

Domain of Influence: Health Care System

Level of Influence: Societal Health Care Policies

Racial disparity in health care utilization and labor market activities could contribute to increasing social costs. Eliminating racial and ethnic disparities is saving billions of dollars and has long-term health promoting consequences. Utilizing the American Community Survey and Minnesota local datasets, Nanney, Myers, Xu, Kent, Durfee and Allen [17] have quantified the monetary cost of racial disparities in the US. Their findings could be used to formulate policies that aim to lower health care and labor market disparities.

### Disentangling Race, Poverty, and Place in Disparities in Physical Activity

Domain of Influence: Sociocultural Environment

Level of Influence: Community Norms/Discrimination

The availability of safe and secure resources to promote healthy lifestyles could have significant impact on both short- and long-term health outcomes of individuals. However, inappropriate social and cultural behaviors toward racial and ethnical minorities in the US could lower the use of health promoting activities, and as a result, poor health outcomes such as obesity and diabetes could emerge. Hawes, Smith, McGinty, Bell, Bower, LaVeist, Gaskin and Thorpe [22] studied the role of race and ethnicity in performing physical activities. They found that black Americans are less likely to use physical health facilities when compared to white Americans.

### No Man is an Island: The Impact of Neighborhood Disadvantage on Mortality

Domain of Influence: Physical-Built Environment

Level of Influence: Community Environment and Resources

Considering influences at the community level, Gaskin et al. [24] quantify the role of neighborhood disadvantage and find that the hazard of dying increased by 9.8% as neighborhood disadvantage increased by one standard deviation. Area-level poverty and mortgage delinquency were important predictors of mortality, even after controlling for individual personal income and occupational status.
